# Vitamin K prophylaxis in newborns

**DOI:** 10.1186/s12887-021-02701-4

**Published:** 2021-09-08

**Authors:** Sophie Jullien

**Affiliations:** Barcelona Institute for Global Health, University of Barcelona, Barcelona, Spain

**Keywords:** Vitamin K deficiency bleeding, Vitamin K prophylaxis, Haemorrhagic disease of newborn, Newborn

## Abstract

We looked at existing recommendations and supporting evidence on the effectiveness of vitamin K given after birth in preventing the haemorrhagic disease of the newborn (HDN).

We conducted a literature search up to the 10th of December 2019 by using key terms and manual search in selected sources. We summarized the recommendations and the strength of the recommendation when and as reported by the authors. We summarized the main findings of systematic reviews with the certainty of the evidence as reported.

All newborns should receive vitamin K prophylaxis, as it has been proven that oral and intramuscular prophylactic vitamin K given after birth are effective for preventing classical HDN. There are no randomized trials looking at the efficacy of vitamin K supplement on late HDN. There are no randomized trials comparing the oral and intramuscular route of administration of prophylactic vitamin K in newborns. From older trials and surveillance data, it seems that there is no significant difference between the intramuscular and the oral regimens for preventing classical and late HDN, provided that the oral regimen is duly completed. Evidence assessing vitamin K prophylaxis in preterm infants is scarce.

## Background

### Introduction

The World Health Organization (WHO) European Region is developing a new pocket book for primary health care for children and adolescents in Europe. This article is part of a series of reviews, which aim to summarize the existing recommendations and the most recent evidence on preventive interventions applied to children under 5 years of age to inform the WHO editorial group to make recommendations for health promotion in primary health care. In this article, we looked at existing recommendations and supporting evidence on the effectiveness of vitamin K given after birth in preventing the haemorrhagic disease of the newborn (HDN).

### Why is vitamin K important?

Vitamin K is required for the synthesis of coagulation factors, being essential for blood clotting. Vitamin K deficiency can lead to excessive and severe bleeding.

### Context

In an infant, vitamin K deficiency can cause bleeding known as HDN or vitamin K deficiency bleeding (VKDB). It can present through three distinct forms: early, classical and late. The early disease occurs within the first 24 h of life and cannot be prevented by prophylactic administration of vitamin K to the newborn. The classical form presents between the days 1 and 7. The late onset HDN occurs between 7 days and 6 months of life, although it is more common between 14 days and 3 months of life, mainly in fully breastfed infants and typically with cutaneous, gastrointestinal or intracranial haemorrhage. At birth, newborns present low level of vitamin K because of the limited placental transfer, a sterile gut, and their immature liver. Therefore, newborns are susceptible to develop HDN. Preterm infants are potentially at higher risk of HDN due to more pronounced hepatic and haemostatic immaturity, and to delayed feeding leading to delay of the gastrointestinal tract colonization involved in the synthesis of the vitamin K [1]. Adequate postnatal vitamin K intake seems therefore crucial in this group of infants. Vitamin K is commonly given as prophylaxis after birth for preventing HDN. Among infants who had not received vitamin K prophylaxis at birth, the incidence of HDN was estimated at 35 (10.5 to 80) per 100,000 live births, being lower in high income countries at 8.8 (5.8 to 17.8) per 100,000 live births [2].

## Key questions

1. Is vitamin K given after birth effective in preventing HDN? (Table 1)
2. Are the oral and parenteral administrations equally effective in preventing HDN?
3. Is one dose versus multiple doses equally effective for preventing the HDN?

**Table 1:** Key questions for vitamin K prophylaxis in newborns

	Key question 1	Key question 2	Key question 3
**Participants**	Neonates		
**Intervention**	Vitamin K supplement	Oral vitamin K supplement	Multiple doses of vitamin K supplement
**Comparison**	Placebo or no vitamin K supplement	Parenteral vitamin K supplement	Single dose of vitamin K
**Outcome**	HDN or VKDB		
**Adverse effects**	Any adverse effect		

Abbreviations: *HDN* Haemorrhagic disease of the newborn, *VKDB* vitamin K deficiency bleeding

## Search methods and selected manuscripts

We described the search methods, data collection and data synthesis in the second paper of this supplement (Jullien S, Huss G, Weigel R. Supporting recommendations for childhood preventive interventions for primary health care: elaboration of evidence synthesis and lessons learnt. BMC Pediatrics. 2021. 10.1186/s12887-021-02638-8).

The search was conducted on the 10th of June 2019, by manual search and by using the search term “vitamin K”. We found three documents from the WHO addressing vitamin K supplementation in newborns. No document was identified from the US Preventive Services Task Force (USPSTF) published recommendations or recommendations in progress. From the PrevInfad workgroup (Spanish Association of Primary Care Pediatrics), we found recommendations on vitamin K prophylaxis for haemorrhagic disease of the newborn, last updated in April 2010. One clinical guideline on postnatal care from the National Institute for Health and Care Excellence (NICE) gives recommendations on prophylactic administration of vitamin K and was therefore included. The search in the Cochrane library returned 38 reviews and four protocols. By screening the titles and abstracts, we included one systematic review and none of the protocols. By looking at the evidence supporting the existing recommendations, we retrieved another Cochrane review. However, as published before 2010, we conducted a search in Pubmed to identify any updated systematic review covering this topic or any relevant trial that was published after the systematic review. The search was conducted on the 28th of June 2019 following the search strategy described in Table 2 and returned 121 manuscripts. By screening titles and abstracts, and after exclusion of duplicates with studies already identified, we included two reviews, two cohort studies, and two position papers from the European Society for Paediatric Gastroenterology Hepatology and Nutrition (ESPGHAN) and from the Canadian Agency for Drugs and Technologies in Health (CADTH).

**Table 2:** Search strategy used in Pubmed

#4 Search (#1) AND #2 Filters: published in the last 10 years	
#3 Search (#1) AND #2	
#2 Search (haemorrhagic disease of the newborn [Title/Abstract]) OR vitamin K deficiency [Title/Abstract]	
#1 Search ((newborn*[Title/Abstract]) OR infant*[Title/Abstract]) OR neonat*[Title/Abstract]	

All the included manuscripts for revision in this article are displayed in Table 3.

**Table 3:** Included manuscripts for revision

Sources	Final selected manuscripts
**WHO**	• WHO recommendations on newborn health [3] • Pregnancy, childbirth, postpartum and newborn care: a guide for essential practice [4] • Recommendations for management of common childhood conditions: evidence for technical update of pocket book recommendations [5]
**USPSTF**	None
**PrevInfad**	• 2010 recommendations and supporting document [6]
**NICE**	• NICE clinical guideline 2015 – Postnatal care up to 8 weeks after birth [7]
**CDC**	• Recommendations [8]
**AAP**	• Policy statement 2003 (reaffirmed in 2020)^a^ [9, 10]
**Cochrane Library**	• Ardell 2018 – Prophylactic vitamin K for the prevention of vitamin K deficiency bleeding in preterm neonates (Systematic review) [11] • Puckett 2000 (published before 2010, but supporting evidence used for PrevInfad and WHO recommendations) [12]
**Pubmed**	• Sankar 2016 (Systematic review) [2] • Martín-López 2011 (Systematic review) [13] • Löwensteyn 2019 (Cohort study) [14] • Witt 2016 (Cohort study) [15] • Mihatsch 2016 (ESPGHAN position paper with review of the literature and recommendations) [16] • Canadian Agency for Drugs and Technologies in Health 2015 (Review) [17]

**Abbreviations:***AAP* American Academy of Pediatrics; *CDC* Centers for Disease Control and Prevention; *ESPGHAN* European Society for Paediatric Gastroenterology Hepatology and Nutrition; *NICE* National Institute for Health and Care Excellence; *PrevInfad* PrevInfad workgroup from the Spanish Association of Primary Care Pediatrics; *USPSTF* US Preventive Services Task Force; *WHO* World Health Organization

^a^The literature search was conducted on 28th June 2019 but the date of the reaffirmation of the AAP statement was added during the review process of this article

## Existing recommendations

We summarized the existing recommendations and the strength of recommendations as per their authors in Table 4.

**Table 4:** Summary of existing recommendations

Source	Ref	Date	General recommendations for use of prophylactic vitamin K in newborns
**WHO**	[3, 5]	2012, updated in 2017	“All newborns should be given 1 mg of vitamin K intramuscularly (IM) after birth (i.e. after the first hour by which the infant should be in skin-to-skin contact with the mother and breastfeeding should be initiated).” (*Strong recommendation, moderate quality evidence*) “Neonates requiring surgical procedures, those with birth trauma, preterm newborns, and those exposed in utero to maternal medication known to interfere with vitamin K are at especially high risk of bleeding and must be given vitamin K (1 mg IM).” (*Strong recommendation, moderate quality evidence*)
	[4]	2015	“Give 1 mg of vitamin K IM to all newborns, one hour after birth”
**PrevInfad**	[6]	2010	After birth, prophylactic vitamin K should be administered to prevent HDN (*Strong recommendation*). After birth, IM administration of 1 mg of vitamin K is recommended to prevent classical HDN (*Strong recommendation*). After birth, IM administration of 1 mg of vitamin K is recommended to prevent late HDN (*Weak recommendation*). In case of parents who do not want IM administration, oral administration of 2 mg of vitamin K is recommended followed by 1 mg weekly until 12 weeks of age in totally or partially breastfed infants (*Weak recommendation*). For preterm babies: • < 32 weeks and > 1000 g: 0.5 mg IM or IV • < 1000 g independently of gestational age: 0.3 mg IM or IV (*Weak recommendation*)
**NICE**	[7]	2015	“All parents should be offered vitamin K prophylaxis for their babies to prevent the rare but serious and sometimes fatal disorder of vitamin K deficiency bleeding.” “Vitamin K should be administered as a single dose of 1 mg intramuscularly as this is the most clinically and cost-effective method of administration.” “If parents decline intramuscular vitamin K for their baby, oral vitamin K should be offered as a second-line option. Parents should be advised that oral vitamin K must be given according to the manufacturer’s instructions for clinical efficacy and will require multiple doses.” Note: These recommendations were established in 2006 when the first clinical guideline was published, but updated in 2015.
**CDC**	[8]	Updated 2018	One shot intramuscularly in the thigh just after birth, can be delayed up to 6 h after birth.
**AAP**	[9, 10]	2003 (updated 2020)	“Vitamin K1 should be given to all newborns as a single, intramuscular dose of 0.5 to 1 mg.” “Additional research should be conducted on the efficacy, safety, and bioavailability of oral formulations and optimal dosing regimens of vitamin K to prevent late VKDB.” “Health care professionals should promote aware- ness among families of the risks of late VKDB associated with inadequate vitamin K prophylaxis from current oral dosage regimens, particularly for newborns who are breastfed exclusively.”
**ESPGHAN**	[16]	2016	“Healthy newborn infants should either receive: (a) 1 mg of Vitamin K_1_ by IM injection at birth, OR (b) 3 × 2 mg Vitamin K_1_ orally at birth, at 4 to 6 days and at 4 to 6 weeks, OR (c) 2 mg Vitamin K_1_ orally at birth, and a weekly dose of 1 mg orally for 3 months.” “The oral route is not appropriate for preterm infants and for newborns who are unwell, have cholestasis or impaired intestinal absorption or are unable to take oral vitamin K, or those whose mothers have taken medications that interfere with vitamin K metabolism.”
**CADTH**	[17]	2015	“Single intramuscular dose of vitamin K (0.5 mg for birthweight ≤1500 g or 1.0 mg for birthweight ≥1500 g) should be administered to all newborns within the first 6 h after birth.” “If intramuscular vitamin K is refused by parents, an oral dose of 2 mg vitamin K was recommended at the time of first feeding, followed by a second dose at 2 to 4 weeks, and a third dose at 6 to 8 weeks.”

**Abbreviations:***AAP* American Academy of Pediatrics; *CADTH* Canadian Agency for Drugs and Technologies in Health; *CDC* Centers for Disease Control and Prevention; *ESPGHAN* European Society for Paediatric Gastroenterology Hepatology and Nutrition; *HDN* haemorrhagic disease of the newborn; *IM* intramuscular; *IV* intravenous; *NICE* National Institute for Health and Care Excellence; *PrevInfad* PrevInfad workgroup from the Spanish Association of Primary Care Pediatrics; *VKDB* vitamin K deficiency bleeding; *WHO* World Health Organization

## Existing evidence

One Cochrane systematic review was identified, addressing prophylactic vitamin K for the prevention of HDN in preterm neonates (Ardell 2018) [11]. We identified another Cochrane review looking at the effectiveness of prophylactic vitamin K in the prevention of HDN in all infants (mainly term babies) (Puckett 2000) [12]. Although it was published prior to 2010, we decided to include this review in this summary as this is the backbone evidence supporting the existing recommendations from both the WHO and PrevInfad. The Puckett 2000 Cochrane review looked at the effectiveness of vitamin K prophylaxis in the prevention of classic and late HDN, but clinical bleeding was only assessed as an outcome for classical HDN. The 13 identified randomized controlled trials (RCTs) or quasi-RCTs included only term infants or infants without complications (this statement was unknown for one trial), and eight of them included only infants who were on exclusive breastfeeding. These studies were published between 1960 and 1998, and the countries where they were conducted were not mentioned. Although authors of the review acknowledge the poor methodological quality of the included trials (randomization not described or incorrect, high losses to follow-up), the certainty of the evidence in this review was not graded. However, the quality of this evidence was graded as moderate to low by the authors of the WHO recommendations for management of common childhood conditions [5].

Another systematic review was identified, with the literature search conducted up to 2008, including the studies included in the Puckett 2000 systematic review and four additional studies that were published after 2000 [13]. The systematic review on vitamin K prophylaxis for preventing HDN conducted by Sankar et al. did not identify any additional trial through the literature search conducted up to 2013 [2].

### Vitamin K versus placebo or no treatment for preventing HDN

#### IM vitamin K (single dose) versus placebo or no treatment

Two RCTs (from the 1960’s) comparing a single dose of IM vitamin K versus placebo or no intervention showed a reduction of clinical bleeding at one to seven days of life (relative risk [RR] 0.73; 95% confidence interval [CI] 0.56 to 0.96; and RD -0.02; 95%CI − 0.04 to 0.00; one trial), and a reduction of bleeding after circumcision (RR 0.18; 95%CI 0.08 to 0.42; and risk difference [RD] -0.11; 95%CI − 0.16 to − 0.07; one trial) [12]. Two other trials (from the 1990’s) looked at the same comparison but reported different outcomes, and showed improved biochemical indices of coagulation status among children who received vitamin K (reduction in the detection of prothrombin induced by vitamin K absence-II [PIVKA II] at 1 to 7 days of life: RR 0.43 [95%CI] 0.26 to 0.71; and RD -0.49 [95%CI 0.70 to − 0.28]; 2 trials) [12].

#### Oral vitamin K (single dose) versus placebo or no treatment

Three trials compared a single dose of oral vitamin K versus placebo or no supplement and showed a significant reduction in the detection of PIVKA II at 3 days of life, favouring vitamin K (RR 0.40 [95%CI 0.26 to 0.61] and RD -0.44 [95%CI − 0.60 to − 0.29]; 3 trials) [12]. Clinical bleeding was not assessed in these studies.

### Oral versus intramuscular vitamin K for preventing HDN

There are no randomized trials comparing the oral and intramuscular (IM) route of vitamin K prophylaxis in newborns. As such trials are unlikely to be conducted, the efficiency of the different regimens is assessed by national surveillance, with the risk of underreporting the true incidence of HDN. Table 5 summarizes the different regimens of vitamin K prophylaxis used in different European countries with the corresponding incidence of HDN from surveillance data [6, 15, 16]. These data highlight the lack of standardized dosages currently used in Europe, and the multiple oral doses used as equivalence of a single IM dose. Mihatsch et al. concluded that while data from older studies suggest that IM may be more effective than the multiple oral doses of vitamin K for preventing late HDN, the more recent data from surveillance systems does not seem to support a significant difference between the IM and the oral route for preventing late HDN [16].

**Table 5:** Vitamin K prophylaxis regimens and associated incidence of HDN from surveillance data in Europe

Country	Vitamin K prophylaxis	Incidence of HDN per 100,000 infants, RR (95% CI)
Denmark		
1994 to June 2000	2 mg po at birth, 1 mg po weekly for 3 months	0.0 (0 to 0.9)
After June 2000	2 mg IM at birth	Not available
France	2 mg po weekly for 6 weeks	Not available
Germany	2 mg po for 3 doses: days 1, 4–10 and 28–42	0.44 (0.2 to 0.9)
Netherlands	(A) 1 mg po at birth, 25 μg po daily from week 2 to 13 (B) 1 mg po at birth, 150 μg po daily from week 2 to 13	(A) 3.2 (1.2 to 6.9) Intracranial HDN, general and targeted surveillance (A) 1.6 (0.4 to 5.1) and 3.1 (1.9 to 5.0) (B) 1.3 (0.5 to 3.2) and 1.2 (0.6 to 2.3)
United Kingdom	1 mg IM at birth	0.1
	2 mg po for 3 doses: day 1, weeks 1 and 4	0.43
Spain	1 mg IM at birth	‘Almost inexistent’^a^
Switzerland	2 mg po for 3 doses: day 1, day 4, week 4	0.0 (0.0 to 0.81)^b^ [16] 1.09 (0.4 to 2.6) [17]

**Abbreviations:***CI* confidence interval; *HDN* haemorrhagic disease of the newborn; *IM* intramuscular; *po* per oral; *RR* relative risk

^a^No statistical data available, but ‘almost inexistent’ in the country when enquired through the Spanish authorities (personal communication)

^b^Between 2005 and 2011, one early and four late cases of HDN were reported out of 458,184 breastfed newborns. Vitamin K prophylaxis was rejected from the parents in four cases, and the fifth newborn only received the first dose of vitamin K prophylaxis. The incidence of HDN shown is for infants who completed the three doses of oral vitamin K prophylaxis [16]

The efficacy of the oral regimen relies on the compliance with the protocol (multiple doses). Incomplete oral prophylaxis have been detected in newborns with HDN receiving the oral regimen [6, 16, 18]. In addition, different formulations of oral vitamin K exist. Therefore, the respective dosage recommendations have to be followed with caution.

With a focus on late HDN and in absence of trials assessing the efficacy of prophylactic vitamin K on late HDN, Sankar et al. also looked at data from surveillance studies [2]. Four studies were found from Germany, the British Isles, Japan and Thailand, from 1981 to 2004. From two studies, 1 mg of IM or subcutaneous vitamin K prophylaxis reduced incidence of late HDN when compared to placebo (RR 0.02; 95%CI 0.00 to 0.10). Oral prophylaxis compared to placebo also reduced incidence of late HDN, although effect was probably lower (reduction by 97 and 80% for IM versus placebo and oral versus placebo respectively, from one study) and no pooled estimate was performed.

Although no studies looked at clinical bleeding as an outcome for assessing direct comparison of oral versus IM administration of vitamin K, some studies evaluated biochemical parameters and are described below.

#### Single dose of oral versus IM vitamin K

Four studies looked at PIVKA II in infants who received a single dose of vitamin K either orally or IM. There were no significant differences on PIVKA II at several endpoints (1 to 7 days, 2 weeks, 1 month and 3 months) from individual nor pooled results [12]. Another study looked at the effect of oral and IM on the combined activity of coagulation factor II, VII and X, prothrombin antigen and PIVKA II, with no significant differences between the two administration routes [13]. Few other studies looked at biochemical parameters without significant differences [13].

#### Multiple doses of oral versus single dose of IM vitamin K

One study compared three doses of oral vitamin K given just after birth, at 7 and 30 days of life with a single dose IM given after birth. Authors investigated levels of plasma vitamin K, being higher in the oral group at 2 weeks (mean difference [MD] 0.80 ng/mL [95%CI 0.34 to 1.27]) and 3 months (MD 0.30 [95%CI 0.10 to 0.50]), but with no differences between the two regimens at 1 month. Authors also investigated the international normalised ratio (INR), finding no significant differences between the two groups at 2 weeks, 1 month and 3 months [12]. Two additional studies found no differences between the two regimens by looking at PIVKA II and the combined activity of coagulation factor II, VII and X in the first 2 months of life, and at prothrombin time [13].

Another study looked at the incidence of HDN in breastfed infants with biliary atresia who received one of the three following regimens of prophylactic vitamin K according to their country and date of birth: (oral 25 μg group) 1 mg orally at birth followed by 25 μg orally daily from week 2 to week 13 of life was given to infants born in the Netherlands from 1991 to 2011, (oral 150 μg group) 1 mg orally at birth followed by 150 μg orally daily from week 2 to week 13 of life was given to infants born in the Netherlands from 2011 to 2015, (2 mg IM group) single dose of 2 mg IM at birth was given to infants born in Denmark from 2000 to 2014 [15]. HDN occurred in 45/55 (82%) infants from the oral 25 μg group, in 9/11 (82%) of the 150 μg group, and in 1/25 (4%) of the 2 mg IM group, leading to the conclusion that an oral prophylaxis of 1 mg of vitamin K followed by a daily oral dose of 25 or 150 μg fails to prevent HDN in breastfed infants with unrecognized biliary atresia, in comparison to a single IM dose [15].

### Vitamin K prophylaxis for preventing HDN in preterm babies

The Ardell 2018 Cochrane review looked at the effect of vitamin K prophylaxis in the prevention of VKDB in preterm infants (gestational age < 37 weeks) [11]. No RCT looking at vitamin K via any route of administration versus no vitamin K was identified. Only one RCT comparing three arms of prophylactic vitamin K (0.5 mg IM, 0.2 mg IM and 0.2 mg IV) in 80 preterm babies under 32 weeks of gestational age was identified [1]. There were no statistically significant differences between 0.2 mg IV versus 0.2 mg IM on bleeding complications (RR 7.00 [95%CI 0.38 to 129.11]), intraventricular haemorrhage > grade II (RR 2.00 [95%CI 0.19 to 20.72]), presence of PIVKA-II at day 5 (RR 1.52 [95%CI 0.37 to 6.23]) or day 25 (RR 1.08 [95%CI 0.07 to 16.36]), necrotizing enterocolitis (RR 1.00 [95%CI 0.15 to 6.57]) and on sepsis (RR 1.00 [95%CI 0.28 to 3.58]). There were no statistically significant differences neither between 0.2 mg IV nor 0.5 mg IM on the same outcomes. When looking at higher (0.5 mg) versus lower (0.2 mg) dose of IM vitamin K, there were also no statistically significant differences with broad 95%CI for the same outcomes. The certainty of the evidence was graded as low for all the outcomes, due to the small sample size from one trial.

### Adverse effects

When the IM route with standard precautions at anterolateral thigh is chosen, the risk of local hematomas, infections and neuromuscular damage at the site of the injection is very low. Although the IM injection is likely to be painful, simple strategies including skin-to-skin, breastfeeding, or administration of glucose/sucrose, are effective to alleviate pain in newborns [16, 19, 20]. The rationale for the substitution of the IM route by an alternative oral route has to be seen from the child rights convention perspective [21]. The association of IM administration of vitamin K with certain forms of childhood cancer or any other severe side effect has been conclusively rejected by several studies [6].

## Summary of findings

- All newborns should receive vitamin K prophylaxis, as it has been proven that oral and intramuscular prophylactic vitamin K given after birth is effective for preventing classical HDN.
- There are no randomized trials looking at the efficacy of vitamin K supplement on late HDN.
- There are no randomized trials comparing the oral and intramuscular route of administration of prophylactic vitamin K in newborns. As such trials are unlikely to be conducted, the efficiency of the different regimens is assessed by national epidemiological surveillance.
- Older trials comparing oral versus intramuscular administration of vitamin K do not report clinical bleeding as outcome but conclude that both routes of administration improve biochemical indices of coagulation status.
- Looking at surveillance data from European countries, it seems that there is no significant difference between the IM and the oral regimens for preventing classical and late HDN, provided that the oral regimen is duly completed.
- The oral route is not appropriate for newborns with biliary atresia.
- Evidence assessing vitamin K prophylaxis in preterm infants is scarce.

## Data Availability

Not applicable.
